# Neurobehavioral Deficits and Increased Blood Pressure in School-Age Children Prenatally Exposed to Pesticides

**DOI:** 10.1289/ehp.0901582

**Published:** 2010-02-25

**Authors:** Raul Harari, Jordi Julvez, Katsuyuki Murata, Dana Barr, David C. Bellinger, Frodi Debes, Philippe Grandjean

**Affiliations:** 1 Corporación para el Desarrollo de la Producción y el Medio Ambiente Laboral, Quito, Ecuador; 2 Department of Environmental Health, Harvard School of Public Health, Boston, Massachusetts, USA; 3 Division of Environmental Health Sciences, Akita University, Akita, Japan; 4 National Center for Environmental Health, Centers for Disease Control and Prevention, Atlanta, Georgia, USA; 5 Department of Neurology, Children’s Hospital, Boston, Massachusetts, USA; 6 Department of Environmental Medicine, University of Southern Denmark, Odense, Denmark

**Keywords:** acetylcholinesterase, blood pressure, maternal exposure, neurotoxicity syndromes, occupational exposure, organophosphorus compounds, pesticides, prenatal exposure delayed effects

## Abstract

**Background:**

The long-term neurotoxicity risks caused by prenatal exposures to pesticides are unclear, but a previous pilot study of Ecuadorian school children suggested that blood pressure and visuospatial processing may be vulnerable.

**Objectives:**

In northern Ecuador, where floriculture is intensive and relies on female employment, we carried out an intensive cross-sectional study to assess children’s neurobehavioral functions at 6–8 years of age.

**Methods:**

We examined all 87 children attending two grades in the local public school with an expanded battery of neurobehavioral tests. Information on pesticide exposure during the index pregnancy was obtained from maternal interview. The children’s current pesticide exposure was assessed from the urinary excretion of organophosphate metabolites and erythrocyte acetylcholine esterase activity.

**Results:**

Of 84 eligible participants, 35 were exposed to pesticides during pregnancy via maternal occupational exposure, and 23 had indirect exposure from paternal work. Twenty-two children had detectable current exposure irrespective of their prenatal exposure status. Only children with prenatal exposure from maternal greenhouse work showed consistent deficits after covariate adjustment, which included stunting and socioeconomic variables. Exposure-related deficits were the strongest for motor speed (Finger Tapping Task), motor coordination (Santa Ana Form Board), visuospatial performance (Stanford-Binet Copying Test), and visual memory (Stanford-Binet Copying Recall Test). These associations corresponded to a developmental delay of 1.5–2 years. Prenatal pesticide exposure was also significantly associated with an average increase of 3.6 mmHg in systolic blood pressure and a slight decrease in body mass index of 1.1 kg/m^2^. Inclusion of the pilot data strengthened these results.

**Conclusions:**

These findings support the notion that prenatal exposure to pesticides—at levels not producing adverse health outcomes in the mother—can cause lasting adverse effects on brain development in children. Pesticide exposure therefore may contribute to a “silent pandemic” of developmental neurotoxicity.

During prenatal development, the brain is particularly vulnerable to neurotoxicity (Andersen et al. 2000; Grandjean and Landrigan 2006); it is critically dependent upon appropriate supplies of essential nutrients, and malnutrition, as reflected by stunted growth, can result in significant neurodevelopmental delays (Grandjean et al. 2006). If the complex developmental processes are disturbed, there is little potential for later repair, and the functional consequences can therefore be permanent (Grandjean and Landrigan 2006; Rice and Barone 2000). Studies of nutritional deficiencies have demonstrated that cardiovascular development may also be affected (Walker et al. 2001).

Acetylcholine is a major synaptic transmitter substance that also serves as a neurotrophic signal during brain development (Slotkin 2004). Experimental studies in rodents suggest that cholinesterase inhibitors that are used as insecticides, such as organophosphates, can interfere with the brain development and lead to permanent damage (Ahlbom et al. 1995; Slotkin 2004). Epidemiologic evidence of the neurodevelopmental toxicity of pesticide exposure during pregnancy is growing (Berkowitz et al. 2004; Bjørling-Poulsen et al. 2008; Engel et al. 2007; Eskenazi et al. 2007; Grandjean and Landrigan 2006; Grandjean et al. 2006; Guillette et al. 1998; Handal et al. 2007, 2008; Rauh et al. 2006; Ruckart et al. 2004; Young et al. 2005). Of particular interest are the studies performed in areas where organophosphates are widely used (Bjørling-Poulsen et al. 2008). The results obtained suggest that developmental pesticide exposure can cause delayed mental development detectable at 6–24 months of age (Eskenazi et al. 2007; Rauh et al. 2006; Whyatt et al. 2004; Young et al. 2005), with reduced motor functions and visual acuity (Handal et al. 2008) and reduced short-term memory and attention being apparent later on (Ruckart et al. 2004). Our own pilot study of school children in Tabacundo, Ecuador, showed visuospatial deficits and increased systolic blood pressure associated with prenatal exposure and increased reaction time at increased levels of current exposure (Grandjean et al. 2006).

Based on a successful pilot study (Grandjean et al. 2006), we chose to carry out a more detailed neurobehavioral study of school children in the floriculture area of Tabacundo-Cayambe in northern Ecuador. The flower industry is a main source of income and the major employer of women of reproductive age. In the local greenhouses, about 30 different pesticides are routinely applied, among which organophosphate insecticides are most widely used. In the present study, we applied the same cross-sectional design as the pilot study (Grandjean et al. 2006), and we substantially supplemented the clinical test battery with validated neurobehavioral instruments sensitive to environmental neurotoxicity and unlikely to be influenced by cultural factors.

## Methods

Located on the Andean plateau north of Quito at an altitude of about 2,800 m, the town of Tabacundo has two public elementary schools and a medical care center operated by the Ministry of Public Health. Eligible subjects included all of the 6- to 8-year-old children in the two lowest grades (called second and third) of one of the two schools. Through the school headmaster, an invitation to participate was extended to the parents, with a written description of the study. One of the teachers served as contact person. An informational meeting for parents was held at the school one evening, and written parental consent was obtained for all eligible children. A 2-hr examination of each child was scheduled at the nearby medical care center during normal school hours or in the late afternoon. The results of the examination were reported to the mother after completion of the study. Skilled personnel interviewed the mothers and completed detailed questionnaires with 150 items about the mother’s own exposure history and demographic parameters and the child’s past medical history and current health. Each interview took 1 hr. The questionnaires were then reviewed by a specialist in public health and occupational medicine, and scores were developed for the standard of family housing (traditional/contemporary, running water, and sewage drainage), nutrition (two or three meals per day and extent of animal protein in the diet), and potential maternal pesticide exposure during pregnancy. Interview and scoring were performed without knowledge of the child’s clinical results. The study protocol was developed in accordance with the Helsinki Declaration and was approved by the Comité de Bioética of the Centro de Biomedicina, Universidad Central del Ecuador.

### Physical examination

A physical examination was conducted by a local pediatrician. The blood pressure was measured under standardized conditions with the child relaxing in a chair. We used a small, child-sized cuff and a standard sphygmomanometer. The average of two measurements (in millimeters mercury) was used. Height (to the nearest 0.5 cm) and weight (to the nearest 0.1 kg) were measured using the routine procedures of the clinic. Both the body mass index (BMI; weight in kilograms divided by height in meters squared) and the ponderal index (weight in kilograms divided by height in meters cubed) were calculated. A capillary blood sample was obtained by a finger stick to determine hematocrit. When indicated, referral was made and treatment initiated.

### Neurophysiologic measures

Visual and auditory evoked potential latencies and audiometric and heart rate measurements were recorded using standard methods published elsewhere (Murata et al. 2005; Nakao et al. 2007). From the heart rate data, we calculated the coefficient of variation of the R-R intervals (CV_RR_) as the ratio (in percent) of the standard deviation of the R-R intervals to the average R-R interval (heart rate). These outcomes were selected because of evidence that they may be affected by developmental exposure to some environmental pollutants (Brucker-Davis 1998; Chiappa 1997; Choi et al. 2009; Morreale de Escobar et al. 2000; Murata et al. 1999).

### Neuropsychological tests

The neuropsychological tests were selected on the basis of their psychometrical validity, sensitivity to the neurotoxicity of environmental pollutants, and relative insensitivity to cultural factors (Grandjean et al. 1997, 1999; Lezak 1995; White et al. 1994).

#### Motor speed and dexterity

On the Finger Tapping Task (Lezak 1995), the child tapped a key for a series of 15-sec sessions, first completing one session with the preferred hand for practice, then two sessions with the preferred hand, and then two more sessions with the nonpreferred hand. We used the standard board for this test (WW-1597-NP; Psychological Assessment Resources, Odessa, FL, USA), but the thickness of the board was increased by 1 cm to allow children with small hands to move the tapping arm effortlessly (Grandjean et al. 1999; Lezak 1995). We used the sum of taps from the two nonpractice sessions with the preferred hand as the best indicator of motor speed and dexterity.

The Santa Ana Form Board, a test of motor coordination, has four rows of square holes into which fit square pegs with a cylindrical head. Half of the circular area on the top of each peg is white, and the other half is black. The subject had to lift each peg and rotate it 180°. We used the time needed to finish the task with the preferred hand as indicator of motor coordination (Grandjean et al. 2006).

#### Attention

In Conners’s Kiddie Continuous Performance Test (K-CPT, version 5), the child was required to press the spacebar (hits) each time a picture appeared, unless the picture was of a ball, during a 7-min test duration (Conners 2001). Scores derived from the test were the total number of missed responses (omissions), false positives (commissions), perseverations (repeated hits), and the overall average reaction time. This test measures vigilance/attention and is appropriate for testing children at early school age.

#### Visuospatial function and memory

In the Copying Test of the Stanford-Binet, 4th edition (Thorndike et al. 1986), visual designs were copied by the child through the use of drawings. This test assesses visuospatial and visuoconstructional function and was negatively associated to prenatal pesticide exposure in the first pilot study (Grandjean et al. 2006). Similar associations were found with methylmercury exposure (Grandjean et al. 1999). In addition to the standard Stanford-Binet Copying Test scores used in the pilot study (Grandjean et al. 2006), we also applied a detailed scoring system of error types to ascertain subtle deficits (Chevrier et al. 2009; Sullivan 1999) both for the entire series (designs 13–28) and for the easier and more age-appropriate designs 13–20. As in the pilot study, we included a memory trial (Stanford-Binet Copying Recall Test), in which the child was asked, after about 20 min, to draw as many of the designs as possible. A scoring scheme appropriate for recognizable designs was employed for the memory condition that used less strict criteria than the standard scoring system (Sullivan 1999). A neuropsychologist, who had scored the Copying Test drawings from a previous cohort of Faroese children and Ecuadorian children in the pilot study (Debes et al. 2006; Grandjean et al. 2006), scored these as well.

#### General intelligence

Raven’s Colored Progressive Matrices (Raven et al. 1979) provide a nonverbal assessment of general intelligence in children 5–11 years of age. It is based on the same principle as the Raven’s Standard Progressive Matrices Test but provides greater discrimination among performances in the lower range. Widely used in cross-cultural settings because of the nonrepresentational stimuli, it assesses the ability to detect an organizing principle in visual materials, requiring the examinee to recognize spatial, design, and numerical relationships (Raven et al. 1979).

#### Short-term auditory memory

We used two tests that assess aspects of short-term memory. The Wechsler Intelligence Scale for Children–Revised (Wechsler 1974) Digit Span Test required that the child repeat strings of digits forward and in reversed order. As in our previous studies of this age group (Grandjean et al. 1999, 2006), only the forward condition was used. We also used a Spanish translation of the Stanford-Binet (Thorndike et al. 1986) Memory for Sentences and Digit String tests. The Memory for Sentences Test provides an assessment of auditory span in a naturalistic context of connected speech. The Digit String Test is similar to the Wechsler’s Digit Span Test.

### Exposure assessment

Previous studies in the floriculture industry in this area (Colosio et al. 2004) have documented that occupational exposures are at low doses and mainly as a consequence of percutaneous uptake. Pesticides are usually applied three or more times every week, but specific occupational safety programs are generally absent. When women become pregnant, they usually do not report it for fear of being fired, and in the absence of paid leave, they usually try to work until close to delivery. Some women worked in a variety of service occupations. Paternal exposures generally involved greenhouse work that included pesticide application and maintenance, including mixing and storage of pesticides. Other paternal employment, mainly in construction trades, did not involve any important neurotoxic exposures.

In the structured maternal interview, which was conducted without knowledge of the clinical findings, systematic information was collected on maternal employment, including exposure conditions and the use of personal protective equipment. The mothers were also asked to provide their social insurance card to confirm their employment and time period in the floriculture company. Questions were also asked regarding possible adverse effects related to the greenhouse work. Potential pesticide exposure was evaluated by an experienced specialist in occupational medicine without knowledge of the clinical data.

The children’s current exposure to pesticides was ascertained by two approaches, as in the pilot study (Grandjean et al. 2006). A blood sample (one drop from finger prick) was obtained to measure erythrocyte acetylcholine esterase (AChE) activity using Test-mate equipment and test solutions from EQM Research, Inc. (Cincinnati, OH, USA). This parameter reflects the combined impact of pesticide exposure on inhibition of AChE activity. Because enzyme recovery is slow, it reflects a longer-term average exposure (Lotti 1995). In addition, for measurement of pesticide metabolites, spot urine samples were collected from the children into wide-mouthed Qorpak collection bottles, which were then immediately capped with the companion Teflon-lined screw cap and labeled. The samples were immediately refrigerated, frozen within 4 hr of collection, and subsequently shipped on dry ice in an insulated container for analysis at the Centers for Disease Control and Prevention (Atlanta, GA, USA). Pesticide metabolite analyses were conducted using an established gas chromatography/tandem mass spectrometry method under tight quality assurance (Bravo et al. 2002). All of the metabolite concentrations were quantified using isotope dilution calibration and evaluated on both a whole-volume and creatinine-adjusted basis. Analytes included major organophosphate breakdown products that occur in urine: three dimethylphosphate metabolites (dimethylphosphate, dimethyldithiophosphate, and dimethylthiophosphate) and three diethylphosphate metabolites (diethylphosphate, diethyldithiophosphate, and diethylthiophosphate). The level of detection was 0.3 ng/L for dimethylphosphate and dimethylthiophosphate and 0.1 ng/L for all of the other alkylphosphate metabolites measured.

### Data analysis

Prenatal pesticide exposure was separated into three category groups: (*a*) no parental exposure, (*b*) paternal exposure only, and (*c*) maternal exposure irrespective of paternal status. Two dummy variables, one for each parental exposure group, were included in the models and compared with the no exposure group category. Stunting was assessed by using the height-for-age *Z*-score, calculated from the anthropometric data of the children and the World Health Organization (WHO) Anthro 1.02 software (WHO Global Database on Child Growth and Malnutrition 2006). As recommended by the WHO, children who had *Z*-scores lower than −2 were considered stunted (de Onis et al. 2000; Semba and Bloem 2001). For children providing a urine sample, a composite indicator of recent pesticide exposure was generated, given that each pesticide yields one type of phosphate metabolite, either dimethyl or diethyl. We therefore summed the molar concentrations of dimethyl metabolites (originating from compounds, e.g., dichlorvos, malathion, and parathion) and diethyl metabolites (from, e.g., chlorpyrifos and diazinon), as described elsewhere (Grandjean et al. 2006; Mage et al. 2008). However, because most children showed a detectable level of only one or a few metabolites, we dichotomized the variable into “not currently exposed” (*n* = 59, all metabolites below the detection level) and “exposed” (*n* = 22, at least one detectable metabolite).

Confounders were identified from *a priori* considerations of relevant factors that might influence nervous system development in this community (Grandjean et al. 2006; Larrea and Kawachi 2005). Age, school grade, and sex were considered obligatory covariates, and the BMI was a mandatory covariate for blood pressure. Other potential cofactors were child’s trauma, other injury, meningitis in the past medical history, current hematocrit, having repeated one school grade, parental education (primary school only or above), current nutrition (number of meals per day), and sociodemographic covariates that comprised race, housing (traditional or other), running water at home, house connected to a central sewer line, number of siblings, marital status, maternal access to health care (i.e., childbirth taking place at home or at the hospital), and maternal smoking and alcohol use during pregnancy. Because this study is based on a small number of subjects, we used the change in estimate > 10% to determine the final selection of covariates. Standard parametric statistical tests were applied for normally distributed outcomes and logistic regressions for outcomes that could not be transformed to normality. A *p*-value ≤ 0.05 was used to determine statistical significance. For generation of binary outcomes in this study, we used the median as the cutoff point. Because the odds ratios (ORs) in the logistic regressions do not approximate relative risks and may tend to be biased away from the null (Spiegelman and Hertzmark 2005), we also attempted to apply log-binomial models as an alternative method, although this was of limited use because of the small sample size. A joint analysis was done by adding the data from 69 participants in the pilot study (Grandjean et al. 2006). In this larger analysis, the highest quintile of the standard score was applied as the cutoff level when analyzing the skewed distribution of the Stanford-Binet Copying Test (designs 13–20) results.

## Results

All children attending Ecuadorian second and third grades were invited to participate in the examinations. However, we excluded one child for a clinical history of meningitis and two more because they were > 9 years of age. Important sociodemographic characteristics are described in Table 1. We found that 35 children had prenatal exposure from their mothers’ work during pregnancy, 23 children were indirectly exposed because of the father’s work during the wife’s pregnancy, and 26 were free from any prenatal exposure due to parental work. None of the mothers reported any adverse effects that could be linked to pesticide exposures in the greenhouses. Characteristics of the children with different prenatal exposure status were similar (*p* > 0.2), except for paternal employment, household salary, traditional housing, and parity. Socioeconomic conditions were better in the prenatally exposed groups, whereas children with and without current exposure had similar socioeconomic characteristics. Children with and without current exposure differed in age, school grade, number of daily meals, paternal employment, and sewage drainage at home (three children did not provide samples for assessment of current exposure). We considered all of these parameters for covariate adjustment.

Table 2 shows the number of children considered currently exposed in relation to history of prenatal exposure. The AChE was inversely associated with the sum of all urinary metabolites (Spearman’s rho = −0.23; *p* = 0.04). However, current exposure, whether based on individual metabolites or groups of metabolites, did not differ between children with and without prenatal exposure.

Table 3 presents the adjusted results in relation with children’s neuropsychological functioning. In crude analyses (data not shown), we found no statistically significant difference between the exposure groups. But after covariate adjustment, the children with prenatal exposure from maternal work showed statistically significant lower scores on four tests [i.e., Finger Tapping Task, Santa Ana Form Board, Stanford-Binet Copying Test (designs 13–20), and the Stanford-Binet Copying Recall Test]. The last parameter also appeared to be affected by paternal exposure during the pregnancy. We obtained similar results for the Stanford-Binet Copying Recall Test outcome from a log-binomial model to estimate the relative risk (data not shown). We found no clear trends for the remaining neurobehavioral outcomes, including the audiometric and neurophysiologic (brainstem evoked potential latencies) tests (data not shown). Further adjustment for the child’s hematocrit, AChE level, and presence of urinary pesticide metabolites did not influence the association between prenatal exposure and outcome scores.

The tests that were associated with prenatal pesticide exposure were also affected by the age of the child. For example, copying errors in Stanford-Binet Copying Test (designs 13–20), the regression coefficient for age (in years) was −0.30. Thus, the prenatal maternal exposure regression coefficient of 0.47 was consistent with a developmental delay of 0.47/0.30 = 1.6 years. Similarly, for the number of taps of Finger Tapping Task, the age coefficient of 3.6 means that the delay associated with maternal exposure (adjusted coefficient = −7.1) was consistent with a developmental delay of 7.1/3.6 = 1.9 years. Reaction time (milliseconds) on the CPT was the only neuropsychological outcome on which the scores of children with current exposure and no exposure appeared to differ, although only with a borderline statistical significance (*p* = 0.098).

Table 4 shows the adjusted results for the children’s clinical outcomes. Again, only maternal prenatal exposure group showed statistically significant associations, for example, a positive association with systolic blood pressure and an inverse association with BMI. The latter also had a significant inverse association with paternal exposure. Associations of maternal and paternal exposure with ponderal index were comparable to those estimated for BMI (data not shown). Heart rate and heart rate variability (CV_RR_) outcomes did not differ between exposure groups.

Some outcomes of the present study were similar to outcomes evaluated in the previous pilot study, notably Stanford-Binet Copying Test and blood pressure, both of which showed a significant association with maternal exposure in both studies. Because information was available on paternal exposure from both studies, the data could be combined in a joint analysis. Because the pilot study had used only the standard scores for the Copying Test (Grandjean et al. 2006), we used this parameter in the joint analysis. The combined results (Table 5) replicated and strengthened the findings. Current exposure to pesticides did not affect these outcomes in either study population (data not shown).

## Discussion

This study used the same overall design and a comparable population sample as a previous pilot study (Grandjean et al. 2006) and replicated the associations observed in that study between prenatal pesticide exposure and visuospatial deficits and increased blood pressure. A joint analysis of the data from both studies confirmed that these outcomes were associated with maternal work during pregnancy, not the father’s work, and not the current exposure to pesticides. In addition, the present study identified lower performance on visual memory and motor tasks as possible effects of prenatal pesticide exposure. A small decrease in BMI was also associated with prenatal exposure. Nutritional parameters such as stunting, current hematocrit levels, number of meals per day, and socioeconomic characteristics did not explain these associations. Neurophysiologic measurements, including evoked potential latencies and heart variability, were not associated with either prenatal or current exposures.

The study design aimed at comparing two groups of children (prenatally exposed vs. nonexposed) with similar backgrounds, except for the maternal history of occupational exposure to pesticides. Three socioeconomic indicators showed better conditions in the exposed group (i.e., higher maternal income, quality of housing, and paternal employment). These differences are meaningful, given the possibility for both parents to be economically active, and they replicate previous observations (Grandjean et al. 2006). The same applies to maternal education, although the absence of a measure of maternal IQ is a limitation. Any residual differences not accounted for by the covariate adjustment would be expected to bias the study toward the null hypothesis, that is, toward finding no associations between prenatal pesticide exposure and children’s neuropsychological functioning. Otherwise, the similarities between the two groups regarding past medical history and all other respects, including current pesticide exposure, would suggest that confounding bias may be limited in this study.

A study with a cross-sectional design and retrospective assessment of prenatal exposure cannot provide information about dose–response relationships or the time of the impact of the exposures. However, the standardized and blinded techniques applied to the maternal reports support the validity of the employment-based classification of exposures. Although a small degree of misclassification cannot be excluded (e.g., because of domestic uses of pesticides), it would be expected to result in an underestimate of the true neurotoxicity of the exposure. Although selection bias is a common problem in cross-sectional studies, it is unlikely in our case, because all eligible children participated in the examinations in both studies (Grandjean et al. 2006). Nevertheless, this problem may not be completely resolved, because children not attending school would be missed. The small sample size of the study is also a clear limitation, but the parallel findings in the two studies and the statistically significant findings are remarkable.

The present study included the same neuropsychological instruments used in the previous study in addition to several supplementary tests (Grandjean et al. 2006). They were carefully selected to be sensitive to developmental neurotoxicity in low doses observed in environmental exposures to pesticides and other pollutants such as methylmercury (Grandjean et al. 1997). In addition, the instruments used were also validated to avoid cross-cultural influences (Grandjean et al. 1999; Lezak 1995; White et al. 1994), as would be appropriate for indigenous and mixed populations in rural areas of developing countries. Thus, although the battery did not provide a complete assessment of all functional domains, major functions were covered by tests that were feasible and showed the anticipated association with age.

The results support the notion that, in this cohort of children, prenatal exposures to pesticides are more harmful than current exposures, thereby confirming previous results of other environmental studies of neurodevelopmental toxicity and the theory of window of vulnerability of central nervous system during uterine life (Grandjean and Landrigan 2006). Paternal exposures during pregnancy showed much weaker associations than did maternal exposures during pregnancy, again in accordance with the findings in other studies (Wigle et al. 2009). Furthermore, the clearest deficits were observed in neuropsychological functions that involved visuospatial scorings, thereby replicating the pilot study that used the same test (Stanford-Binet Copying Test), as well as the findings of other studies of organophosphate exposures during pregnancy (Grandjean et al. 2006; Guillette et al. 1998). An association that corresponds to a delay of almost 2 years is large when considering the rapid development in children at early school age.

Moreover, in this case we also observed alterations to memory and motor functions that were in consonance with the findings of previous studies in younger children (Guillette et al. 1998; Handal et al. 2008). In addition, a study on long-term neuropsychological dysfunctions of school-age children exposed to organophosphates pesticides during infancy (Ruckart et al. 2004) reported that both motor inhibition and verbal learning were impaired in the exposed children.

Although nutritional deficiencies may negatively affect neurodevelopment, the associations observed between prenatal pesticide exposure and children’s test scores were independent of stunting and other indicators of nutritional status. Consequently, as previously observed, pesticide exposures and child malnutrition, as common problems in developing countries, may independently increase the risks of long-term neuropsychological impairment (Grandjean et al. 2006).

We did not find associations between pesticide exposure and other neurophysiologic outcomes such as visual and auditory brainstem evoked potentials and audiometric and heart variability examinations. Recent scientific literature has shown that these outcomes are sensitive to neurotoxicants, such as lead and methylmercury (Murata et al. 1999; Rothenberg et al. 2000). These associations have been documented in fairly large studies, and the absence of an association with pesticide exposure could be due to the small sample size in the present study. At least these outcomes appeared less sensitive to pesticide toxicity at the levels of exposure experienced by the children in this cohort.

Regarding blood pressure, we replicated the results of the pilot study (Grandjean et al. 2006). Prenatal exposure was associated most closely with increased systolic blood pressure. This association was independent of relevant covariates, such as stunting and maternal smoking during pregnancy, known as risk factors for increased blood pressure in children (Brion et al. 2008; Morley et al. 1995; Wilson et al. 1998). This observation is consistent with the hypothesis that the autonomic nervous system may play a role with organophosphate toxicity, as has been considered in previous poisoning studies (Bardin et al. 1994).

A decreased BMI was independently related to prenatal organophosphate exposure, a finding that is consistent with some reports from birth cohort studies of reduced birth weight and birth length as an effect of pesticide exposure (Whyatt et al. 2004). There appears to be no report on older children, however. The biological mechanism is unknown, but acetylcholine may stimulate contraction of the uterus, thus resulting in a reduced birth length and weight (Whyatt et al. 2004). BMI in this case may then reflect a long-term consequence. Nevertheless, the percentage of stunting in the study group was higher (38%) than the average percentage estimated for Ecuador in 2004 (25%). Factors other than malnutrition, such as the high altitude of the study area or the Andean genetic background, may contribute to the high calculated proportion of stunted children (Grandjean et al. 2006; Larrea and Kawachi 2005).

The present study suggests that the current level of protection may well be adequate to avoid pesticide toxicity in the worker herself but insufficient to prevent lasting adverse effects in the offspring. Deficits associated with prenatal pesticide exposure may contribute to a “silent pandemic” of developmental neurotoxicity (Grandjean and Landrigan 2006), and this study therefore adds to the evidence suggesting a need for improved control of occupational exposures that may cause intrauterine neurotoxicity (Julvez and Grandjean 2009). Regarding pregnant women at work, conventions on maternity protection of the International Labour Organization (ILO) require that a pregnant woman not be obliged to perform work that has been determined to be harmful to her health or that of her child (ILO 2000). However, the most recent version of this convention has been ratified by only 17 countries so far, and the general practice in Ecuador is for expecting mothers to continue work until the very last day before childbirth; the rules for maternity leave provide no protection against developmental neurotoxicity.

## Figures and Tables

**Table 1:** Sociodemographic and past medical history characteristics of 84 study participants in relation to history of prenatal pesticide exposure and detectable levels of current pesticide exposure.

		Prenatal exposure			Current exposure^a^	
Parameter	All (*n* = 84)	None (*n* = 26)	Paternal only (*n* = 23)	Maternal (*n* = 35)	No (*n* = 59)	Yes (*n* = 22)
Children’s characteristics						
Sex, male (%)	58	65	52	57	61	59
Age [years (median)]	7.1	6.9	7.3	7.3	7.2	6.9
Race, indigenous (%)	32	31	26	37	32	36
School grade, 2 (%)	48	54	52	40	42	68
Repeating school grade, yes (%)	7	11	4	6	8	4
No. of daily meals, 3 (%)	89	92	83	91	90	95
Other characteristics						
Median maternal age (years)	29	29	29	29	29	28
Civil status, married (%)	51	46	43	60	52	45
Parity, > 2 (%)	48	42	65	40	49	36
Smoking during pregnancy, yes (%)	6	8	4	6	7	0
Drinking alcohol during pregnancy, yes (%)	40	27	48	46	41	41
Delivery, at home (%)	42	42	48	37	42	36
Mothers ever used pesticides outside of work (%)	37	35	35	40	41	27
Maternal level of education, < primary (%)	39	35	52	34	42	27
Paternal level of education, < primary (%)	22	16	27	23	20	22
Paternal employed, yes (%)	94	85	96	100	97	86
Salary, 75th percentile (dollars)	180	120	200	160	190	150
Housing, traditional yes (vs. contemporary) (%)	40	58	48	23	39	41
Sewage drainage at home, yes (%)	50	61	39	49	46	63
Drinking water supply at home, yes (%)	80	73	74	89	78	86

a *n* = 81 (three missing samples).

**Table 2:** Number of subjects with detectable levels of current pesticide exposure^a^ (urinary metabolites) among 81 Ecuadorian school children according to prenatal maternal occupational pesticide exposure.^b^

	Prenatal maternal exposure status		
Analyte	Unexposed (*n* = 46)	Exposed (*n* = 35)	*p*-Value
Dimethyldithiophosphates	0/0	0/0	—
Dimethylthiophosphates	7/39	8/27	0.40
Dimethylphosphates	4/42	3/32	0.98
Diethylthiophosphates	3/43	1/34	0.45
Diethyldithiophosphates	0/0	0/0	—
Diethylphosphates	4/42	2/32	0.61
All dimethyl metabolites	8/38	9/26	0.39
All diethyl metabolites	5/41	3/32	0.73
All metabolites	12/34	10/25	0.80

*p*-Values determined for chi-square test for difference in percentages.

a The current pesticide exposure refers the presence of pesticide metabolites in spot urine samples above the limit of detection for each metabolite.

b Results were similar for nonexposed children and those with paternal exposure only, and they were therefore combined as a control group.

**Table 3:** Multivariate analysis results^a^ for prenatal and current exposures to pesticides as predictors of neuropsychological outcomes in Ecuadorian schoolchildren 6–8 years of age.

	Prenatal exposure			Current exposure	
Outcome scores	None [mean (95% CI)^b^ or OR^c^; *n* = 26]	Paternal only [β (95% CI)^b^ or OR (95% CI)^c^; *n* = 23]	Maternal [β (95% CI)^b^ or OR (95% CI)^c^; *n* = 35]	No [mean (95% CI)^b^ or OR^c^; *n* = 59]	Yes [β (95% CI)^b^ or OR (95% CI)^c^; *n* = 22]
Simple motor speed functions (Finger Tapping Task)					
Mean of number of taps, preferred hand^b^	34.4 (19.9 to 48.9)	−3.8 (−9.6 to 2.0)	−7.1 (−12.5 to −1.6)**	34.3 (19.9 to 48.8)	−2.5 (−7.3 to 2.4)

Motor coordination functions (Santa Ana Form Board)					
Mean duration, preferred hand (sec)^c^	1	2.51 (0.49 to 12.97)	5.32 (1.03 to 27.62)**	1	0.76 (0.20 to 2.84)

Attention functions (Continuous Performance Test)					
Reaction time (msec)^b^	680.9 (429.4 to 932.5)	31.7 (−67.3 to 130.7)	20.4 (−73.8 to 114.7)	640.3 (406.6 to 874.0)	64.7 (−12.4 to 141.7)*
No. of omissions^b^	23.8 (5.2 to 42.5)	−2.1 (−9.4 to 5.2)	−3.2 (−10.2 to 3.8)	17.5 (0.3 to 35.0)	4.4 (−1.4 to 10.1)
No. of commissions^c^	1	2.06 (0.31 to 13.7)	3.55 (0.60 to 20.96)	1	1.01 (0.25 to 4.11)
No. of perseverations^c^	1	1.59 (0.33 to 7.77)	0.77 (0.18 to 3.34)	1	2.90 (0.74 to 11.30)

Short-term auditory memory functions					
Wechsler Intelligence Scale for Children-Revised					
Digit Span Test^b^,^d^	2.0 (0.1 to 3.9)	−0.3 (−1.0 to 0.5)	−0.5 (−1.2 to 0.2)	2.1 (0.3 to 3.9)	−0.4 (−1.0 to 0.2)
Stanford-Binet					
Digit String Test^b^,^d^	4.1 (3.0 to 5.2)	−0.0 (−0.4 to 0.4)	−0.2 (−0.6 to 0.2)	4.1 (3.0 to 5.1)	−0.2 (−0.5 to 0.2)
Memory for Sentences Test^b^,^d^	0.9 (−4.8 to 6.5)	0.2 (−2.0 to 2.5)	−0.5 (−2.6 to 1.6)	1.5 (−3.9 to 6.9)	−0.6 (−2.3 to 1.2)

Visual-performance functions					
Raven Test					
Total score^b^	15.7 (9.1 to 22.3)	−0.5 (−3.1 to 2.0)	−1.9 (−4.4 to 0.5)	16.1 (9.6 to 22.6)	−0.6 (−2.7 to 1.6)
Stanford-Binet Copying Test					
No. of errors in copying (designs 13–20)^b^,^e^	0.9 (−0.4 to 2.1)	0.3 (−0.2 to 0.8)	0.5 (0.2 to 1.0)**	1.0 (−0.2 to 2.3)	0.1 (−0.3 to 0.5)
Total errors in copying^c^	1	2.05 (0.39 to 10.65)	2.13 (0.42 to 10.91)	1	1.74 (0.41 to 7.44)

Visual memory function (Stanford-Binet Copying Recall Test)					
No. of correct recalls^c^	1	13.35 (1.75 to 101.93)**	6.62 (1.02 to 42.93)**	1	0.97 (0.25 to 3.82)

a Each row shows results of the two multivariate models (linear or logistic regressions) controlled for the child’s sex, age, BMI, number of daily meals (only in current exposure), stunting, hematocrit, school grade, having repeated one grade, maternal education level, family living in a traditional house, drinking water supply, and paternal education and employment. The nonexposed group was used as the reference category. Mutual adjustment for prenatal and current exposures did not affect the results.

b Normally distributed outcome, linear regression models were used.

c Because the distribution was different from normal, logistic regression models were used to calculate the odds for having a test score below the median.

d Raw score, the coefficient interpretation should be made by comparing them with the means of the reference groups.

e Square root (outcome scores) to accomplish normal distribution. Linear regression models were used. The coefficient interpretation should be made by comparing them with the means of the reference groups.

* *p* < 0.10;

** *p* ≤ 0.05.

**Table 4:** Multivariate analysis results^a^ for prenatal and current exposures to pesticides as predictors of clinical outcomes and heart variability in Ecuadorian school children.

	Prenatal exposure			Current exposure	
Anthropometrics and clinical results	None [mean (95% CI)^b^ or OR^c^; *n* = 26]	Paternal only [β (95% CI)^b^ or OR (95% CI)^c^; *n* = 23]	Maternal [β (95% CI)^b^ or OR (95% CI)^c^; *n* = 35]	No [mean (95% CI)^b^ or OR^c^; *n* = 59]	Yes [β (95% CI)^b^ or OR (95% CI)^c^; *n* = 22]
Weight (kg)^b^	22.5 (19.9 to 25.1)	−1.4 (−3.0 to 0.2)*	−0.7 (−2.1 to 0.8)	21.8 (19.2 to 24.3)	0.8 (−0.6 to 2.3)
Height (cm)^b^	110.9 (105.6 to 116.3)	1.0 (−2.4 to 4.5)	2.1 (−0.9 to 5.2)	111.8 (106.6 to 116.9)	2.2 (−0.8 to 5.2)
Stunted^c^	1	1.04 (0.30 to 3.62)	0.56 (0.18 to 1.76)	1	0.52 (0.17 to 1.64)
BMI^b^	18.0 (16.4 to 19.7)	−1.4 (−2.4 to −0.4)**	−1.1 (−2.0 to −0.2)**	17.3 (15.6 to 18.9)	0.0 (−1.0 to 1.0)
Hematocrit^b^	44.1 (41.7 to 46.6)	1.2 (−0.4 to 2.7)	−0.3 (−1.4 to 1.3)	43.9 (41.6 to 46.1)	1.1 (−0.2 to 2.4)
Blood pressure^b^					
Systolic	90.0 (83.6 to 96.4)	1.2 (−2.9 to 5.3)	3.6 (−0.1 to 7.2)**	91.9 (85.7 to 98.0)	0.4 (−3.1 to 3.9)
Diastolic	55.2 (48.6 to 61.9)	1.5 (−2.7 to 5.8)	2.9 (−1.0 to 6.6)	56.5 (50.2 to 62.8)	1.0 (−2.6 to 4.7)
Cardiac parameters^b^					
Heart rate (per min)	70.9 (60.0 to 81.8)	−2.5 (−9.5 to 4.6)	1.7 (−4.5 to 7.9)	70.0 (59.9 to 80.1)	3.8 (−1.9 to 9.6)
Heart rate variability [CV_RR_ (%)]	6.7 (3.7 to 9.7)	1.0 (−1.0 to 2.9)	0.0 (−1.6 to 1.7)	6.8 (4.0 to 9.6)	0.5 (−1.1 to 2.1)

a Each row shows two multivariate models (linear or logistic regressions) controlled for the child’s sex, age, race, BMI, and stunting. The models with weight, height, stunted, and BMI as outcomes are adjusted only for child’s sex, age, and race. The nonexposed group was used as the reference category. Mutual adjustment for prenatal and current exposures did not affect the results.

b Normally distributed outcome, linear regression models were used.

c Because the distribution was different from normal, logistic regression models were used to calculate the odds of being stunted, that is, *Z*-score below −2.

* *p* < 0.10;

** *p* ≤ 0.05.

**Table 5:** Results with inclusion of pilot study observations (*n* = 69) in multivariate regression analysis of prenatal exposures to pesticides as a predictor of adverse effects in Ecuadorian school children 6–8 years of age.

	Prenatal exposure		
Outcome scores	None [mean (95% CI)^a^ or OR^b^; *n* = 42]	Paternal only [β (95% CI )^a^ or OR (95% CI)^b^; *n* = 41]	Maternal [β (95% CI )^a^ or OR (95% CI)^b^; *n* = 70]
Visual-performance functions (Stanford-Binet Copying Test)^c^			
Standard scores (designs 13–20)^a^	1.8 (−0.1 to 3.7)	−0.3 (−1.0 to 0.4)	−0.7 (−1.3 to −0.1)**
Standard scores (designs 13–20)^b^	1	1.72 (0.47 to 6.27)	6.11 (1.62 to 23.04)#

Blood pressure^a^,^d^			
Systolic	111.8 (107.2 to 116.5)	−1.3 (−4.5 to 1.9)	3.3 (0.5 to 6.1)**
Diastolic	73.7 (68.9 to 78.5)	0.4 (−2.9 to 3.7)	2.5 (−0.4 to 5.5)*

The nonexposed group was used as the reference category. Mutual adjustment for prenatal and current exposures did not affect the results.

a Normally distributed outcome, linear regression models were used.

b Logistic regression models were used to calculate the odds for being worse than the 80th percentile.

c Adjusted for child’s sex, age, number of daily meals, BMI, stunting, school grade, having repeated one grade, maternal age, maternal education level, parity, delivery at home, family living in a traditional house, drinking water supply, sewage drainage at home, and cohort. The groups did not show significant differences in total standard score of Stanford-Binet Copying Test (data not shown).

d Adjusted for child’s sex, age, race, BMI, stunting, and cohort.

* *p* < 0.10;

** *p* ≤ 0.05;

# *p* < 0.01.
